# Combinatorial Engineering of Dextransucrase Specificity

**DOI:** 10.1371/journal.pone.0077837

**Published:** 2013-10-18

**Authors:** Romain Irague, Laurence Tarquis, Isabelle André, Claire Moulis, Sandrine Morel, Pierre Monsan, Gabrielle Potocki-Véronèse, Magali Remaud-Siméon

**Affiliations:** 1 Université de Toulouse; INSA, UPS, INP, LISBP, Toulouse, France; 2 CNRS, UMR5504, Toulouse, France; 3 INRA, UMR792 Ingénierie des Systèmes Biologiques et des Procédés, Toulouse, France; Oak Ridge National Laboratory, United States of America

## Abstract

We used combinatorial engineering to investigate the relationships between structure and linkage specificity of the dextransucrase DSR-S from *Leuconostoc mesenteroides* NRRL B-512F, and to generate variants with altered specificity. Sequence and structural analysis of glycoside-hydrolase family 70 enzymes led to eight amino acids (D306, F353, N404, W440, D460, H463, T464 and S512) being targeted, randomized by saturation mutagenesis and simultaneously recombined. Screening of two libraries totaling 3.6.10^4^ clones allowed the isolation of a toolbox comprising 81 variants which synthesize high molecular weight α-glucans with different proportions of α(1→3) linkages ranging from 3 to 20 %. Mutant sequence analysis, biochemical characterization and molecular modelling studies revealed the previously unknown role of peptide ^460^DYVHT^464^ in DSR-S linkage specificity. This peptide sequence together with residue S512 contribute to defining +2 subsite topology, which may be critical for the enzyme regiospecificity.

## Introduction

Bacterial glucansucrases (EC. 2.4.1.) are transglucosidases that synthesize high molecular weight α-glucans, oligosaccharides or glucoconjugates from sucrose, a low cost agroresource, as glucosyl donor. Depending on their specificity, glucansucrases (GS) catalyze the formation of both linear and branched α-D-glucans with various types of osidic linkages, namely α(1→2); α(1→3); α(1→4) and/or α(1→6) glucosidic bonds. These enzymes are thus attractive tools for glycodiversification due to their ability to produce carbohydrates of diverse size, structure and physico-chemical properties [1].

The GS produced by lactic acid bacteria of the genera *Leuconostoc*, *Streptococcus*, *Lactobacillus, Exigobacterium* and *Weissella* are classified into the family 70 of Glycoside-Hydrolases (GH70) [1–3]. To date, 57 GS enzymes have been biochemically characterized and three-dimensional structures are available for only four glucansucrases [4–9]. These structures were obtained by crystallization of recombinant truncated forms of GS from *Lb. reuteri* 180 (GTF180-ΔN; PDB:3LK), *S. mutans* (GTF-SI; PDB: 3AIE), *Ln. mesenteroides* NRRL B-1299 (ΔN_123_-GBD-CD2; PDB: 3TTQ) and *Lb. reuteri* 121 (GTFA-ΔN; PDB: 4AMC). These four enzymes present different linkage specificity but share a common U-type fold, organized into five domains (A, B, C, IV and V). 

**Figure 1:**
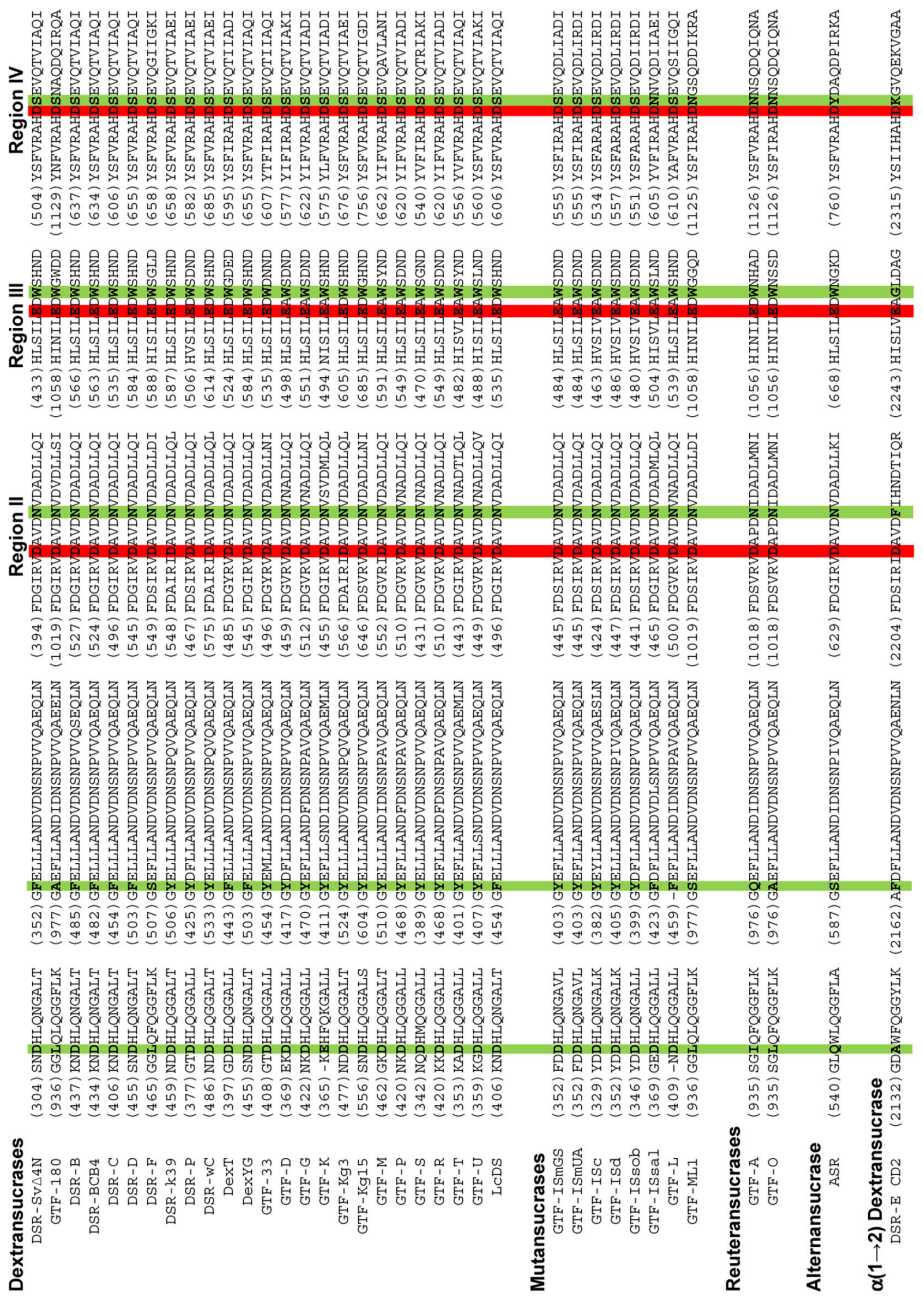
Alignment of GH70 amino acid sequences in the regions chosen for the combinatorial site-directed mutagenesis of DSR-S vardel Δ4N. Sequences are clustered according to the specificity of the glucansucrases. Catalytic (D400 and E438, DSR-S vardel Δ4N numbering) and transition state stabilizer (D511, DSR-S vardel Δ4N numbering) amino acids are highlighted in red; amino acids chosen for mutagenesis are highlighted in green.

All the domains except domain C, are built up from discontinuous segments of the polypeptide chain. The catalytic domain A in GH70 enzymes adopts a (β/α)_8_ barrel fold, which is circularly permuted relative to the related (β/α)_8_ barrel of GH family 13 and 77 enzymes (belonging to the same GH-H clan as family GH70). The active site is shaped as a groove in which a pocket accommodates the glucosyl unit of sucrose in subsite -1 (according to Davies’s subsite numbering [10]). There are no -2 or -3 subsites in GH70 family enzymes, and it has been suggested that they catalyze glucosyl transfer through an α-retaining double displacement mechanism comparable to that of GH family 13 enzymes. The available crystal structures are consistent with this mechanism in which the amino acids D400, E438 and D511 (DRS-S vardel Δ4N numbering, Figure 1) may play the role of the nucleophile, the acid/base catalyst and the transition-state stabilizer, respectively [11,12]. Structural analyses and site-directed mutagenesis experiments also indicate that linkage specificity is probably controlled by the topology of the acceptor subsites, in particular the +1 and +2 subsites. Indeed, several studies highlight the critical role of residues in the conserved regions surrounding the catalytic residues. In particular, mutations of the amino acids downstream from the transition state stabilizer modify the linkage specificity of dextransucrase, mutansucrase, reuteransucrase and alternansucrase [11,13–20]. Numerous chimeric glucansucrase structures have been produced, and display specificities different from those of their parent enzymes, indicating that other regions, for example the extremities of the B-domain, may also contribute to linkage specificity [21,22]. 

The determinants of GS specificity have thus not been completely described. We therefore used an approach based on the construction of a structurally guided library of glucansucrases, derived from one single parent enzyme, to isolate mutants synthesizing high molecular weight α-glucans with various proportions of α(1→3) and α(1→6) linkages [21]. We previously developed a straightforward, sensitive and quantitative NMR-based method for detecting mutants showing new linkage specificity at a throughput of 480 enzyme mutants screened per day [23]. A library of 3.6.10^4^ *E. coli* clones expressing mutants of dextransucrase DSR-S vardel Δ4N, a GS highly specific for the formation α(1→6) glucosidic linkages, have been screened and 303 clones producing enzymes with altered specificity were identified. Seven of these mutants producing dextran polymers with a degree of α(1→3) linkages ranging from 3 to 20 % have been studied in more detail. The dextran products were characterized and differences in size, conformation, as well as ability to form film were described [24]. 

To continue this study, we investigated the structural features of the DSR-S vardel Δ4N mutants that may govern linkage specificity. The semi-rational libraries, from which mutants able to produce dextrans with various proportions of α(1→3) linkages were isolated, was therefore analysed. The tolerance of the targeted amino acid positions to mutations was evaluated. The kinetic properties of the seven mutants producing innovative α(1→3) branched dextran structures were characterized. Combined with their three-dimensional structure models, these findings provide insight into the structural determinants involved in GS substrate recognition and linkage specificity. 

## Materials and Methods

### Sequence alignment

AlignX (Vector NTI Advance 10, Invitrogen) was used to align the sequences of the glucansucrases from *Lb. reuteri* 180 GTF-180 (GenBank accession no. AAU08001.1), *Ln. citreum* NRRL B-1299 DSR-B (GenBank accession no. AAB95453.1), *Ln. mesenteroides* NRRL B-1299CB4 DSR-BCB4 (GenBank accession no. ABF85832.1), *Ln. citreum* B-1355 DSR-C (GenBank accession no. CAB76565.1), *Ln. mesenteroides* Lcc4 DSR-D (GenBank accession no. AAG61158.1), *Ln. citreum*B/110-1-2 DSR-F (GenBank accession no. ACY92456.2), *W. cibaria* LBAE-k39 DSR-k39 (GenBank accession no. ADB43097.3), *Ln. mesenteroides* IBT-PQ DSR-P (GenBank accession no. AAS79426.1), *W. cibaria* CMU DSR-wC (GenBank accession no. ACK38203.1), *Ln. citreum* KM20 DexT (GenBank accession no. ACA83218.1), *Ln. mesenteroides* 0326 DexYG (GenBank accession no. ABC75033.1), *Lb. parabuchneri* 33 GTF-33 (GenBank accession no. AAU08006.1), *S. mutans* GS 5 GTF-D (GenBank accession no. AAA26895.1), *S. gordonii* *str. Challis substr*. CH 1 GTF-G (GenBank accession no. AAC43483.1), *S. salivarius* ATCC 25975 GTF-K (GenBank accession no. AAA26896.1), *Lb. fermentum* Kg3 GTF-kg3 (GenBank accession no. AAU08008.1), *Lb. sakei* Kg15 GTF-kg15 (GenBank accession no. AAU08011.1), *S. salivarius* ATCC 25975 GTF-M (GenBank accession no. AAC41413.1), *S. sanguinis* ATCC 10556 GTF-P (GenBank accession no. BAF43788.1), *S. downei* MFE 28 GTF-S (GenBank accession no. AAA26898.1), *S. oralis* ATCC 10557 GTF-R (GenBank accession no. BAA95201.1), *S. sobrinus* B13N /OMZ176 GTF-T (GenBank accession no. AAX76986.1), *S. sobrinus* B13N GTF-U (GenBank accession no. BAC07265.1), *Ln. citreum* HJ-P4 LcDS (GenBank accession no. BAF96719.1), *S. mutans* GS5 GTF-I_SmGS_ (GenBank accession no. AAA88588.1), *S. mutans* UA159 GTF-I_SmUA_ (GenBank accession no. AAN58705.1), *S. criceti* GTC242/ HS-6 GTF-I_Sc_ (GenBank accession no. BAF62338.1), *S. downei* Mfe 28 GTF-I_Sd_ (GenBank accession no. AAC63063.1), *S. sobrinus* ATCC 33478 / OMZ176 GTF-IS_sob_ (GenBank accession no. BAA02976.1), *S. salivarius* ATCC 25975 GTF-IS_sal_ (GenBank accession no. AAA26896.1), *S. salivarius* ATCC 25975 GTF-L (GenBank accession no. AAC41412.1), *Lb. reuteri* ML1 GTF-ML1 (GenBank accession no. AAU08004.1), *Lb. reuteri* 121 GTF-A (GenBank accession no. AAU08015.1), *Lb. reuteri* ATCC 55730 GTF-O (GenBank accession no. AAY86923.1), *Ln. citreum* B-1355 ASR (GenBank accession no. CAB65910.2), *Ln. citreum* NRRL B-1299 DSR-E (GenBank accession no. CAD22883.1) and DSR-S vardel Δ4N.

### Bacterial strains, plasmids and growth conditions

One shot® *E. coli* TOP 10 (Invitrogen) was used for mutant library construction. One shot® *E. coli* BL21 AI™ (Invitrogen) was used for library selection, screening and larger-scale production of the selected variants. Plasmid pBAD harboring a gene encoding DSR-S vardel Δ4N fused to thioredoxin and polyhistidine tags [25] was used as the template for library construction. Bacterial strains were grown on LB medium supplemented with 100 µg.ml^-1^ ampicillin at 37°C.

### DNA manipulations

Restriction endonucleases and DNA-modifying enzymes were purchased from New England Biolabs and used according to the manufacturer’s instructions. Plasmid DNA was isolated with Qiaprep® Spin miniprep kits (Qiagen). PCR products were extracted from agarose gel with Quiquick® gel extraction kits (Qiagen). DNA sequencing was carried out using the dideoxy chain termination procedure [26] by GATC Biotech SARL (Mulhouse, France). 

### Generation of DSR-S vardel ∆4N mutant libraries

An AatII restriction site was first incorporated into the *dsr-s vardel Δ4N* gene, at position 1881, by inverse PCR introducing a silent mutation using pBAD *Thio*-*dsrs vardel Δ4N*-*His* DNA as the template (Table S1). Then, an 1130 bp cassette, corresponding to positions 829 to 1958 of the *dsr-s vardel Δ4N* gene, was amplified by PCR with primers for_DSRS_Alpha3 and rev_DSR-S_Alpha8 (Table S1). Five µg of purified PCR product was digested with 1 U of DNaseI in the supplied buffer at 20°C in a final volume of 50 µL. The reaction was stopped after 3 min by adding 15 µL of 0.5 M EDTA and heating for 10 min at 75°C. Fragments were separated on 2 % agarose gel and those of 50-100 bp were extracted. Gene reassembly was carried out with approximately 100 ng of purified fragments and 2 µM of degenerated oligonucleotides (Table S1), to mutate positions D306, F353, N404 and W440 for library LibA, and D460, H463, T464, S512 for library LibB. The reaction mixture (30 µL), containing 1 U Phusion® High-Fidelity DNA Polymerase (Finnzyme) in the appropriate buffer and 0.4 mM of each dNTP, was thermocycled according to the following program: one denaturation step at 98°C for 30 s; 40 cycles composed of a denaturation step at 98°C for 10 s, six successive hybridization steps separated by 4°C each, from 65 to 41°C for 10 s each, and an elongation step at 72°C for 20 s; and finally a 2 min step at 72°C. The fully recombined cassettes were isolated from the reassembly products in a last amplification by nested PCR using the primers for_K7a3_nted and rev_K7a8_nted. The purified nested PCR products were digested with the restriction enzymes SpeI and AatII and ligated into the pBAD *Thio-dsrs vardel Δ4N-His* to replace the parental cassette. The ligation products were precipitated by adding 5 volumes of absolute ethanol and the DNA pellet was rinsed twice with 70 % ethanol. The resuspended plasmids were used to transform *E. coli* TOP 10 electrocompetent cells and plated on LB agar supplemented with ampicillin (100 µg.ml^-1^). The transformants were grown overnight at 37°C and the colonies were scraped from the plates for plasmid extraction to constitute the DNA libraries.

### Selection of active glucansucrase mutants

The DNA libraries were used to transform chemically competent *E. coli* BL21 AI cells and plated onto LB agar supplemented with ampicillin (100 µg.ml^-1^). After overnight growth at 37°C, colonies were scraped off, resuspended in physiological water and diluted to an OD_600nm_ of 5.10^-5^. The clones were subjected to selection pressure by plating the suspensions on 22x22 cm plates containing solid M9 mineral medium (42 mM Na_2_HPO_4_, 22 mM KH_2_PO_4_, 18.7 mM NH_4_Cl, 8.5 mM NaCl, 2.5.10^-2^ mM CaCl_2_, 1 mM MgSO_4_) supplemented with ampicillin (100 µg.ml^-1^), 0.02 % arabinose (wt/vol) and sucrose (50 g.L^-1^) as the sole carbon source. The plates were incubated for 7 days at 20°C to allow sufficient expression of recombinant genes encoding active glucansucrases and thus cell growth. This high-throughput selection was carried out at the Laboratoire d’Ingénierie des Systèmes Biologiques et des Procédés (Toulouse, France) with the equipment of the ICEO facility, devoted to the engineering and screening of new and original enzymes.

### Screening for glucansucrase mutants specificity

Active polymerase-positive mutants (identified as colonies covered by a polymer bubble) were picked and transferred into 96-well microplates (Nunc™ Brands Products, Roskilde, Denmark) containing 250 µL LB per well supplemented with ampicillin (100 µg.ml^-1^) using a Biomek 2000 pipettor (Beckman Coulter, Brea, CA). After overnight growth at 30°C under horizontal shaking at 250 rpm, 50 µL of each of these starter cultures was used to inoculate 96-deep-well plates (ABgene, Epsom, UK) containing 500 µL auto-inducing media ZYM-5052 [27] supplemented with ampicillin (100 µg.ml^-1^) and 0.1 % arabinose (w/v). The cultures were grown for 48 h at 20°C in an incubator-shaker (INFORS HT, Bottmingen, Switzerland). The plates were centrifuged (5 min, 3 700 g, 4°C) and the supernatants removed. Bacterial cell pellets were resuspended in 200 µL of lysozyme solution (0.5 mg.ml^-1^), incubated for 20 min at 37°C and frozen at -80°C for 12 h. After thawing for 1 h at room temperature, 800 µL of reaction mixture containing 292 mM sucrose and 50 mM isomalto-oligosaccharides (1 kDa on average, Pharmacosmos, Denmark) in buffered deuterium water (NaAc 50 mM, CaCl_2_ 0.05 g.L^-1^, pH 5.2) was added to each well. Enzymatic reactions were allowed to proceed for 48 h at 25°C under agitation at 700 rpm. The primary structures of the products synthesized were determined by the NMR-based screening method described previously using the flow injection system (Bruker BioSpin GmbH) combining a Bruker Avance 600 MHz spectrometer with a Gilson Liquid Handler [23]. 

### Production and purification of the wild-type enzyme and mutants with altered specificities

*E. coli* BL21 AI cells carrying the pBAD plasmids encoding the Thio-DSR-S vardel ∆4N-His and selected tagged variants were grown for 24 h at 20°C, in flasks containing ZYM-5052 medium supplemented with ampicillin (100 µg.ml^-1^) and 0.1 % arabinose (w/v). The cultures were centrifuged (4 500 g, 15 min, 4°C) and the cell pellets were resuspended to a final OD_600nm_ of 80 in 50 mM sodium acetate buffer, pH 5.2, containing 0.05 g.L^-1^ of CaCl_2_. The suspensions were sonicated and centrifuged (15 000 g, 30 to 60 min, 4°C), and the supernatants were harvested for subsequent purification of the enzymes. The enzymes were purified by affinity chromatography on Probond™ Nickel-Chelating resin (Invitrogen) according the protocol previously described for Thio-DSR-S vardel Δ4N-His purification [25]. Protein concentrations were determined by spectrometry using a NanoDrop® ND-1000 spectrophotometer (Thermo Fisher Scientific, Waltham, MA). 

### Glucansucrase activity assays

One unit of glucansucrase activity corresponds to the amount of enzyme that catalyzes the formation of 1 µmol of fructose per minute at 25°C in 50 mM sodium acetate buffer, pH 5.2, containing 0.05 g.L^-1^ of CaCl_2_ and 100 g.L^-1^ sucrose. The concentration of reducing sugars was determined using the dinitrosalicylic acid method [28], using fructose as the standard.

Kinetic studies were performed using various initial sucrose concentrations from 10 to 600 mM. The reactions were initiated by addition of the enzyme to a final concentration of 0.25 U.mL^-1^. At regular time intervals, samples were withdrawn and immediately heated at 95°C for 5 min to stop the reaction. The concentration of released fructose was determined by ion exchange chromatography, using a Dionex system equipped with an Aminex HPX-87C Carbohydrate column (300 x 7.7 mm; Bio-rad, Hercules, CA, USA), with ultrapure water as eluent at a flow rate of 0.6 mL.min^-1^. The temperature of the column oven was set at 80°C. The initial velocities were calculated directly from fructose production rates and are expressed in µmol of fructose produced per minute and per gram of enzyme. The SigmaPlot “Enzyme kinetics” module (version 3.1) was used for curve fitting of the data and determination of kinetic parameters. 

### α-glucan synthesis

Cell-free extract preparations of the parental Thio-DSR-S vardel Δ4N-His enzyme and selected tagged variants were incubated in sodium acetate buffer 50 mM, pH 5.2, supplemented with 0.05 g.L^-1^ of CaCl_2_ and 292 mM sucrose. Polymer syntheses were carried out at 25°C using 1 U.mL^-1^ of enzyme. Sucrose depletion was monitored by HPLC (see above). Reactions were stopped by a 5 min incubation at 95°C. 

### HPLC analysis

α-glucan concentrations in the reaction media were determined by High-Performance Size Exclusion Chromatography (HPSEC) using Shodex OH-Pack SB-805 and SB-802.5 columns in series. Carbohydrates were eluted using a 0.45 M NaNO_3_ and 1 % (v/v) ethylene glycol solution as the eluent at a flow rate of 0.3 mL.min^-1^[25]. The column oven temperature was set at 70°C. 

Glucose, fructose and leucrose concentrations were determined by ion exchange chromatography using an Aminex HPX-87C Carbohydrate column as described above. The percentages of glucosyl moieties incorporated into free glucose (%G_glucose_) and leucrose (%G_leucrose_), when all sucrose was consumed, were calculated as follows: 

%G_glucose_= [glucose_tf_]/([sucrose_t0_]x180/342) x 100 and %G_leucrose_ = [leucrose_tf_]/[sucrose_t0_] x 100 where [sucrose_t0_] is the initial massic substrate concentration, and [glucose_tf_] and [leucrose_tf_] to the final massic concentration of glucose and leucrose at the end of the reaction.

### The percentages of glucosyl moieties incorporated into High Molecular Weight (HMW) α-glucans were determined as follows

%G_HMW glucans_= [HMW glucans_tf_]/([sucrose_t0_]x162/342) x 100 where [HMW glucans_tf_] correspond to the concentration of HMW α-glucans synthesized at the end of the reaction, and determined on HPSEC chromatogram from the RI signal integration of the peak corresponding to HMW α-glucans. 

The proportion of glucose incorporated into Intermediate Molecular Weight (IMW) α-glucans (gluco-oligosaccharides of DP from 3 to 37) cannot be quantified directly by chromatography due to the weak and disperse signals. Consequently, it was determined as follows:

%G_IMW glucans_= 100 - %G_HMW glucans_ - %G_glucose_ -%G_leucrose_

### NMR spectroscopy

Freeze-dried polymer samples (15 mg) were exchanged twice with 99.9 atom % D_2_O, lyophilized and dissolved in 600 μL of D_2_O. 1D ^1^H NMR spectra were recorded on a BrukerAvance 500 MHz spectrometer using a 5 mm z-gradient TBI probe at 298 K, an acquisition frequency of 500.13 MHz and a spectral width of 8012.82 Hz. The ^1^H-signal from D_2_O was used for automatic lock and a gradient shimming was performed with each sample. Before Fourier transformation, the FIDs were multiplied by an exponential function with a line broadening of 0.3 Hz. Spectra were processed with a 64 k zero filling, baseline correction and were referenced using the TSP-d4 signal at 0 ppm. All NMR data were acquired and processed using TopSpin 2.1 software. The various signals were assigned as previously described [15,16,29–31]. The percentages of α(1→3) and α(1→6) linkages in α-glucans were calculated from the relative intensities of the corresponding anomeric proton signals by integration of peak areas.

### Three-dimensional molecular modeling

The sequence of DSR-S vardel Δ4N catalytic domain (region N119 to K973) was submitted to the I-TASSER server [32–34], an online service for automated protein structure and function prediction, using as restraint the structure of the *Lactobacillus reuteri* N-terminally truncated glucansucrase GTF180 (PDB accession code: 3KLK). The 3D model of DSR-S vardel Δ4N predicted by I-TASSER was then further refined using the CFF91 force-field implemented in the DISCOVER module of the Insight II software suite (Accelrys, San Diego, CA, USA). For the minimization, the CFF91 cross terms, a harmonic bond potential, and a dielectric of 1.0 were used. An initial minimization with a restraint on the protein backbone was performed using a steepest descent algorithm followed by conjugated gradient minimization steps until the maximum RMS was less than 0.5. In a subsequent step, the system was fully relaxed.

A 3D model of the D460M:H463Y:T464M:S512C mutant was constructed by introducing, *in silico*, the divergent amino acids in this mutant into the corresponding positions in the parental enzyme, using the Biopolymer module of the Insight II software package (Accelrys). The conformations of the mutated residue side chains were optimized by manually selecting a low-energy conformation from a side-chain rotamer library. Steric clashes (van der Waals overlap) and non-bonded interaction energies (Coulombic and Lennard-Jones) were evaluated for the various side-chain conformations. The mutant model was subsequently minimized following the same energy minimization procedure as described for the parental enzyme.

Sucrose was manually docked into the active site of DSR-S vardel Δ4N and the D460M:H463Y:T464M:S512C mutant using the structure of the *Lactobacillus reuteri* N-terminally truncated glucansucrase GTF180 in complex with sucrose (PDB code: 1HZ3) as a model. Complexes were then optimized using the procedure above described. 

One of the putative products of the D460M:H463Y:T464M:S512C mutant, the α-D-Glcp-(1→6)[α-D-Glcp-(1→3)]α-D-Glcp-(1→6)-D-Glc*p* tetrasaccharide, was docked into the mutant enzyme active site using the automated flexible docking program FlexX (Biosolveit) [35,36]. The tetrasaccharide molecule was covalently docked into the active site using covalent constraint with catalytic residue D400. All parameters were set to the standard values as implemented in Version 3.1.1. The docking region was defined to encompass all amino acids in the protein for which at least one heavy atom was located within a sphere of 6.5 Å radius around the center of mass of catalytic D400. For each docking, the top 30 solutions, those with the best FlexX scores, were retained. All drawings were performed using PyMOL software (DeLano Scientific).

## Results and Discussion

### Selection of amino acid targets

A semi rational approach was implemented to alter DSR-S vardel Δ4N linkage specificity. Amino acids presumed to be involved in linkage specificity were first identified by sequence alignment of 37 GS catalytic domains (Figure 1). In this alignment were included 25 dextransucrases exhibiting mainly α(1→6) linkage specificity, eight mutansucrases specific for α(1→3) linkages, two reuteransucrases catalyzing both α(1→4) and α(1→6) linkage synthesis [37,38], one alternansucrase generating alternated α(1→3)/α(1→6) linkages [39], and the sequence of the second catalytic domain of DSR-E (DSR-E CD2), specific for α(1→2) glucosidic linkage synthesis [39–41]. Five highly conserved segments emerged from the alignment, three of them corresponding to the signature regions II, III and IV of the GH13 family, and which are close to the catalytic residues. Arbitrarily, sequence divergences in these highly conserved sequences were considered to be relevant to linkage specificity and the corresponding positions were selected for mutation. 

Within segment S304-T314 (DSR-S vardel Δ4N numbering, Figure 1), residue D306 is well conserved amongst dextransucrases. However, this position is occupied by a glutamine in ASR and an alanine in DSR-E CD2. In reuteransucrases, leucine or isoleucine are found. Interestingly, a leucine residue is also found at this position in DSR-F, a dextransucrase synthesizing 1 % of α(1→4) linkages [42]. Downstream from this region, in the segment G352-N374, all the dextransucrases that are highly specific for α(1→6) linkages, except DSR-F and GTF-180, contain an aromatic residue (phenylalanine or tyrosine) at position 353. DSR-F, GTF-180, reuteransucrases and alternansucrase have an alanine, a serine or a glutamine residue at this position. In the signature regions II and III, residues N404, downstream from the catalytic nucleophile D400, and W440, upstream from the general acid/base catalyst E438, are strictly conserved among GH70 glucansucrases, except in DSR-E CD2 which displays α(1→2) linkage specificity. The residues corresponding to N404 and W440 in GH13 enzymes interact with the substrate aglycon [43]. In GTF-180 and GTF-A, they are parts of the subsites +1 and +2 for substrate (sucrose) and acceptor (maltose) molecules [5,9]. Consequently, we considered the residues D306, F353, N404, and W440 likely to be important for linkage specificity. We also included residue S512 in the analysis, because as a part of the tripeptide^512^SEV^514^, it is located immediately downstream from the second aspartic acid of the catalytic triad in the motif IV and has been reported to affect linkage specificity [11,15–17,19,20].

Three other positions, located in a non-conserved region of GH70 enzymes, emerged as possibly significant from structural analysis of GTF180-ΔN glucansucrase in complex with maltose (PDB code: 3KLL) [5]. In this structure, four residues corresponding to D460, H463, T464 and S512 of DSR-S vardel Δ4N interact with maltose in subsite +2, either directly or through water molecule mediation. It has been suggested that these residues participate in the correct positioning of the acceptor for driving the formation of α(1→6) linkage [5]. By analogy, these residues were assumed to participate in linkage specificity and were thus selected as targets for mutation. 

Consequently, engineering efforts were focused on eight residues: D306, F353, N404, W440, D460, H463, T464 and S512. The ISOR method [44] was used to generate two libraries named “LibA”, targeting amino acids D306, F353, N404, W440, and "LibB", targeting amino acids D460, H463, T464 and S512. The synthetic oligonucleotides used for saturation mutagenesis were designed to contain the conventional degenerated NNS codons at the targeted positions and to give theoretically access to the 20 possible amino acid residues (Table S1). 

### Analysis of initial library

**Figure 2:**
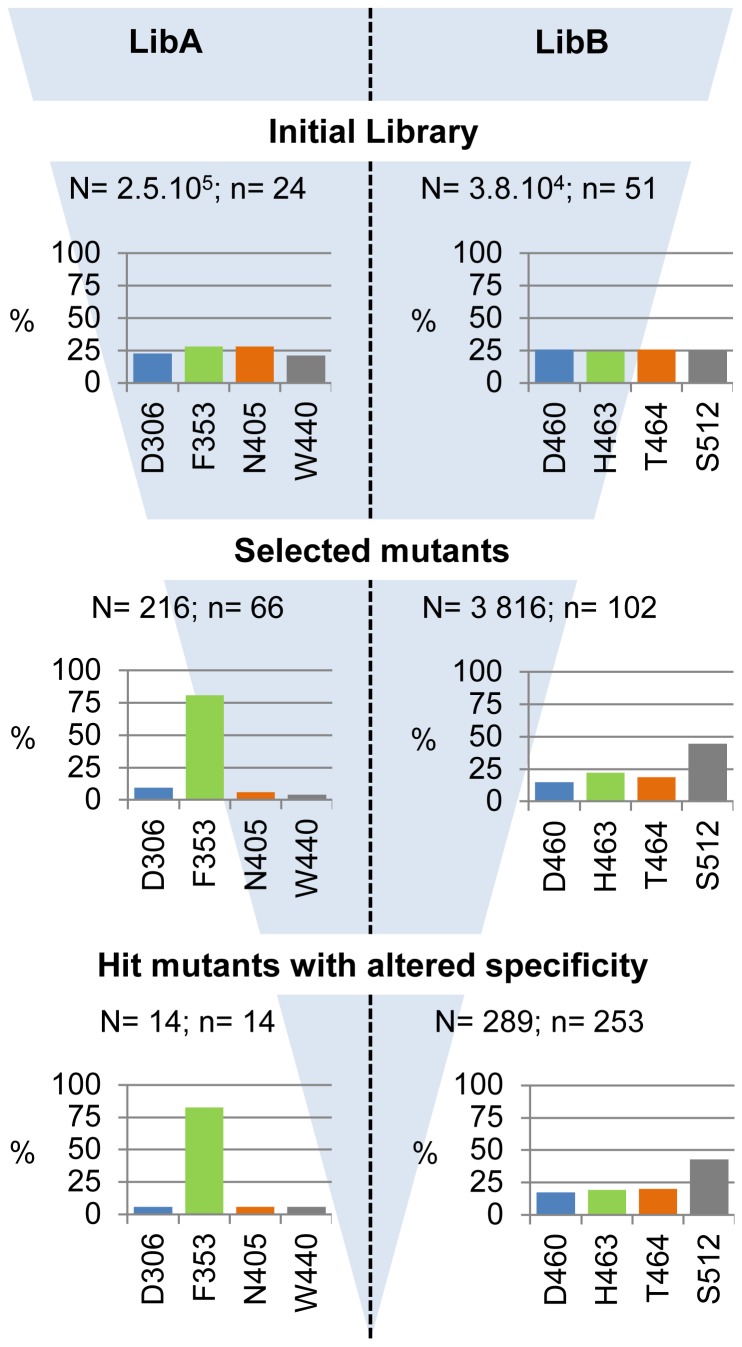
Sequence analysis of the LibA and LibB mutants and evolution of the mutation frequency at each step of the screening protocol. The numbers N and n correspond to the number of isolated clones and sequenced mutants at each step, respectively.

A total of 2.5.10^5^ and 3.8.10^4^ clones were obtained for LibA and LibB, respectively. 24 and 51 clones were randomly isolated from LibA and LibB, respectively, and sequenced (Figure 2). All the sequences corresponded to mutant enzymes. In addition, all the 24 sequences of LibA mutants differed from one another. In LibB, only three of the 51 mutants were found duplicates. Thus, both libraries exhibited a very low level of redundancy. For both libraries, an average of 3 mutations per protein sequence was observed. Mutations were incorporated at all targeted positions and with generally similar frequencies (Figure 2). This provides evidence that the ISOR method was effective for both generating and combining mutations between distant or close residues, such as those found in LibA and LibB, respectively. 

### Analysis of the mutant libraries after primary screening

DNA was isolated from libraries LibA and LibB and used to transform *E. coli* BL21 AI cells. A total of 1.5.10^4^ clones for LibA and 2.1.10^4^ clones for LibB were plated on selective sucrose medium [45]. Only clones producing active glucansucrase mutants can grow on this medium, and form a polymer bubble if the expressed mutant retains polymerase activity. This step, consisting of positive selection coupled to visual screening, led to the isolation of 216 clones from LibA, fewer than 2 % of the clones selected, expressing GS mutants with polymerase activity (Figure 2). Sequence analysis of the 66 mutants revealed that 80 % of the mutations affected position 353, the other 20 % being equally distributed between the three other positions. This indicates that positions 306, 404 and 440 are less tolerant to mutations than position 353. Possibly, there is more stringency at these positions for substrate recognition and/or transglucosylating activity. 

From LibB, 3,816 clones exhibiting polymerase activity were detected (20 % of the mutants selected). Mutational spectrum analysis (Figure 2) revealed that 44 % of the mutations were at position 512, showing that residue S512 is more permissive to mutations than residues D460, H463 and T464.

### Analysis of the mutants *with* altered linkage specificity

**Figure 3:**
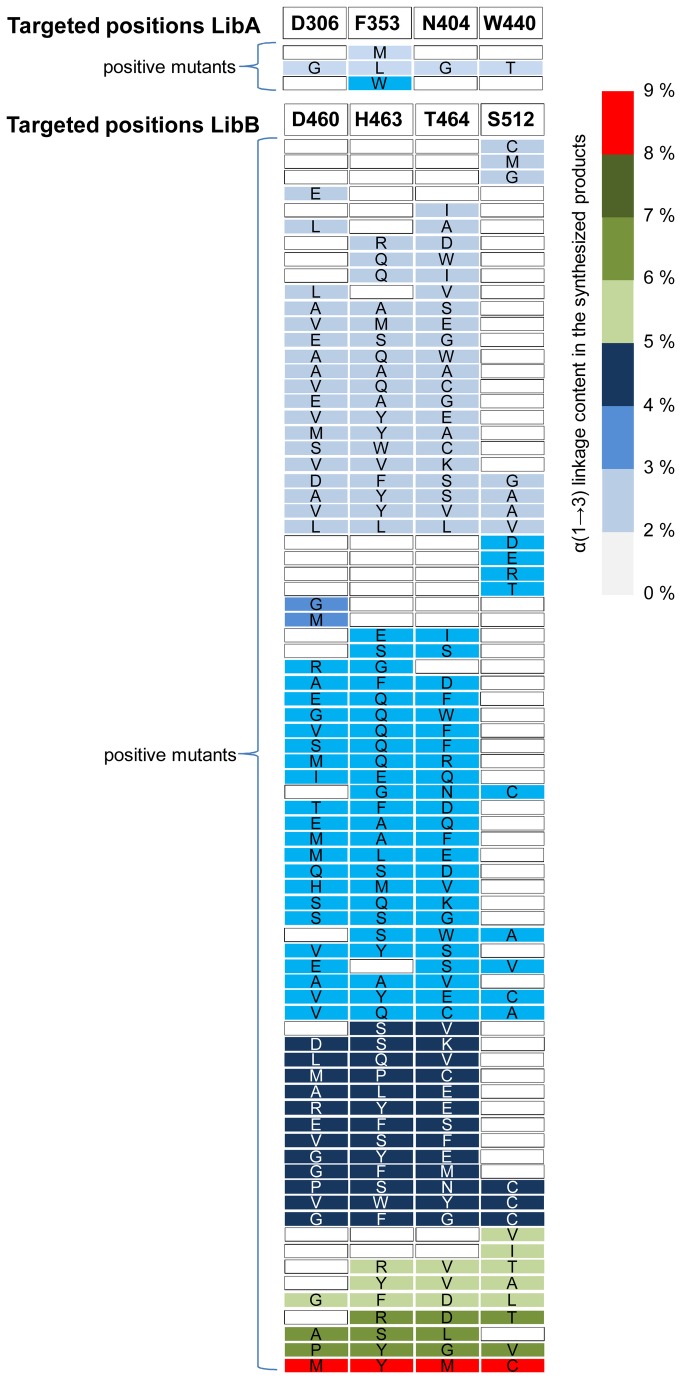
Mutants with altered specificity identified by NMR-based screening.

The 4,032 clones expressing mutants with polymerase activity were subjected to the high-throughput NMR-based screening method previously described [23]. Briefly, the method is based on the 1D ^1^H NMR detection of the α(1→3) and α(1→6) linkages in the gluco-oligosaccharides (GOS) synthesized by each mutant, in the presence of sucrose as a glucosyl donor and isomalto-oligosaccharides (IMOS) as acceptor. This IMOS corresponds to a population of small dextrans with an average molecular mass of 1 kDa and an average degree of polymerization of 7. This step allowed us to identify 14 clones from LibA and 289 from LibB expressing GS with osidic linkage specificity that was different to that of the parental enzyme. Sequencing of these 303 mutants revealed that the distribution of mutations was similar to that determined from sequencing of the polymerase-active mutants after primary screening (Figure 2). Substantial sequence redundancy was observed as only 3 and 78 different mutants (at the protein sequence level) were sorted out from LibA and LibB, respectively (Figure 3). Furthermore, all three mutants from LibA displayed mutations at position 353, highlighting the role of this position in subsite +1 for positioning acceptors during transglucosylation. Mutants synthesizing GOS with the highest level of α(1→3) linkage were retrieved from LibB (Figure 3). Almost all the LibB mutants synthesizing GOS with more than 5 % of α(1→3) linkages exhibited a mutation at position 512. This is consistent with the role previously attributed to the residues downstream from the transition state stabilizer (D511) in the control of linkage specificity [11,15–17,19,20]. Mutant D460M:H463Y:T464M:S512C was the “best hit”, with an ability to incorporate more than 8 % of α(1→3) linkages in the synthesized GOS. The linkage specificity of the single mutant S512C was not substantially different from that of the wild type enzyme. In contrast, 10 triple mutants (12 % of the mutants with altered specificity), displaying mutations at positions 460, 463 and 464, synthesized GOS with 4 times more α(1→3) linkages than the wild type enzyme (Figure 3). These findings demonstrate the importance of recombination in remodeling the active site topology and controlling linkage specificity. 

This preliminary analysis led us to study seven mutants (F353T; S512C; F353W; H463R:T464D:S512T; H463R:T464V:S512T; D460A:H463S:T464L and D460M:H463Y:T464M:S512C) in more detail. These mutants were used to produce α-glucans from sucrose only. Mostly HMW α-glucans are synthesized in these reaction conditions. 1D ^1^H NMR analyses of the various products showed that the proportion of α(1→3) linkages was comprised between 3 to 20 % (Table 1). The physico-chemical properties of these polymers have been described elsewhere and, here, we report further analysis of the relations between the mutations and the catalytic properties of these mutants. 

**Table 1:** Products synthesized by the parental *Thio*-DSR-S vardel Δ4N-*His* enzyme and the seven selected mutants, from 292 mM sucrose, as determined by HPSEC and 1D ^1^H NMR spectroscopy.

**Enzyme**	Product distribution (%)*				**Elution yield (%)**	**α-glucan linkages (%)^a^**	
	HMW glucan	IMW glucan	Leucrose	Glucose		α(1→3)	α(1→6)
*Thio*-DSR-S vardelΔ4N-*His*	61	31	6	2	96	4	96
F353T	34	36	23	7	75	3	97
S512C	69	19	9	3	89	6	94
F353W	48	23	22	7	100	9	91
H463R:T464D:S512T	54	32	9	5	97	12	88
H463R:T464V:S512T	56	31	8	5	90	15	85
D460A:H463S:T464L	54	37	6	3	99	15	85
D460M:H463Y:T464M:S512C	54	32	7	7	99	20	80

* Product distribution describes the relative amount of glucosyl units from sucrose incorporated into the different products.

^a^ Data from [24].

### Relations between mutations, linkage specificity and catalytic properties

**Figure 4:**
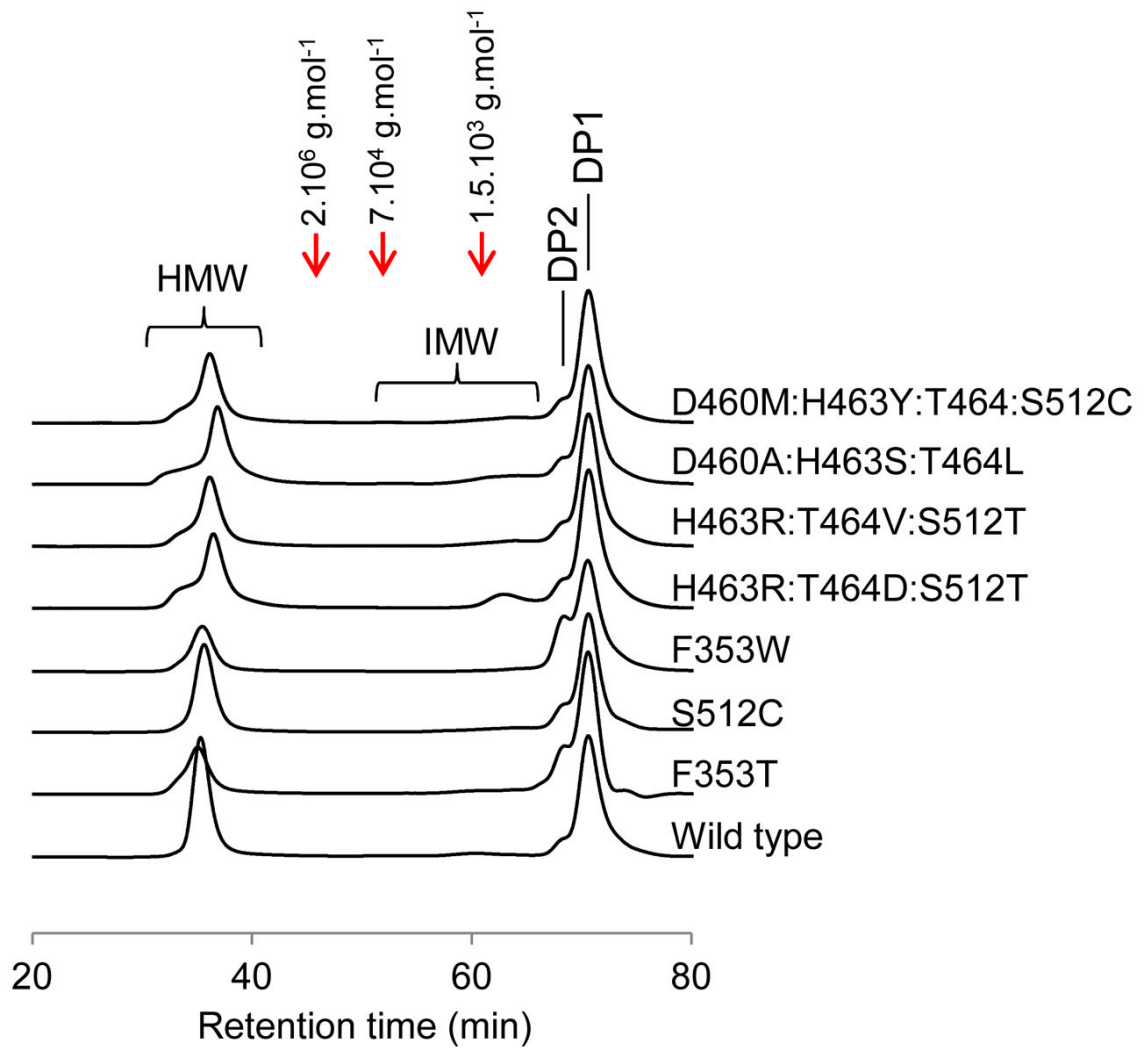
HPSEC chromatograms of products synthesized by *Thio*-DSR-S vardel Δ4N-*His* and the seven selected mutants, from 292 mM sucrose. HMW: High Molecular Weight α-glucans; IMW: Intermediary Molecular Weight α-glucans; DP2: disaccharides; DP1: monosaccharides. Red dotted lines indicate the retention time of standard dextrans.

The lowest α(1→3)/α(1→6) linkage ratio was for HMW α-glucans synthesized by mutants exhibiting only a single mutation. The mutants that synthesized HMW α-glucans with more than 10 % of α(1→3) linkages harbored three or four mutations, and the highest proportion of α(1→3) linkages (20 %) being obtained by the quadruple mutant D460M:H463Y:T464:S512C. To our knowledge, the role of these residues has not previously been explored by glucansucrase engineering. The reaction products were also subjected to HPSEC analysis (Figure 4). Like parental enzyme, all the mutants produced two populations of α-glucans referred as HMW α-glucans (molar mass higher than 10^7^ g.mol^-1^) and IMW glucans (molar mass in the range 0.5.10^3^ to 5.7.10^3^ g.mol^-1^). The HMW α-glucans produced by these seven mutants were previously shown to have various structures [24]. The seven mutants also catalyzed the formation of leucrose, glucose and fructose (Table 1). Except for the two single mutants F353T and F353W, the product yields for the mutants are only slightly affected, with respect to the parental enzyme, suggesting that the mutations at positions D460, H463, T464, and S512 do not greatly alter acceptor binding ability. However, mutants F353T and F353W displayed a 3 to 4-fold higher leucrose yield, suggesting that the mutations may favor fructose binding and thereby result in greater leucrose formation. 

Mutants D460M:H463Y:T464M:S512C, D460A:H463S:T464L, H463R:T464D:S512T and F353T displayed a 10 to 30-fold reduction of catalytic efficiency (Table 2). For the first three of them, this was mainly due to a decrease of the *k*_*cat*_ values whereas for the F353T mutant, the loss of catalytic efficiency is mainly due to a drastic increase of the *K*_*m*_ value, about 15-times higher than that of the parental enzyme. Interestingly, F353T mutant also exhibits a 76-fold increase of *K*_*m*_ compared to the F353W mutant. The aromatic ring of phenylalanine or tryptophan residues at position 353 is likely to be critical for binding the fructosyl ring in subsite +1. Kinetic analyses revealed that, as previously shown for the parental enzyme [11], five variants —F353T, S512C, H463R:T464D:S512T, D460A:H463S:T464L and D460M:H463Y:T464M:S512C— were inhibited by sucrose excess. By contrast, there was no such inhibition of mutants F353W and H463R:T464V:S512T. Notably, mutant H463R:T464V:S512T, that synthesizes HMW α-glucans with 15 % α(1→3) linkages, is slightly more efficient than the parental enzyme. Moreover, at a substrate concentration of 600 mM, the initial velocity of H463R:T464V:S512T is 3 times higher than that of DSR-S vardel Δ4N. In contrast, mutant H463R:T464D:S512T, differing only by the nature of the amino acid substitution at position 464, is eight times less efficient than the parental enzyme. This reveals a critical role for this position. 

**Table 2:** Kinetic properties of seven mutants with altered specificity, and of the wt *Thio*-DSR-S vardel Δ4N-*His* (values are means of three independent assays).

**Enzyme**	***K*_*m*_ (mM)**	***K*_*s*_ (mM)**	***k*_*cat*_ (s^-1^)**	***kcat*/*K*_m_ (s^-1^.mM^-1^)**
*Thio*-DSR-S vardelΔ4N-*His*	7.75*	326*	584*	75.4
F353T	115.8±nd	46.6±nd	338.5±nd	2.9
S512C	3.9±1.0	405.8±65.9	207.1±9.7	53.1
F353W	1.5±0.1	-	86±1.2	57.3
H463R:T464D:S512T	15.5±1.4	201.7±17.7	172.1±6.7	11.1
H463R:T464V:S512T	6.9±0.2	-	610.2±2.7	88.4
D460A:H463S:T464L	5.3±0.5	552.7±54.8	43.9±0.9	8.3
D460M:H463Y:T464M:S512C	15.5±2.8	175.1±36.7	38.2±3.2	2.5

* Data from [25].

### 3D- modeling of DSR-S catalytic domain

A 3D-model of the DSR-S vardel Δ4N catalytic domain was generated from the structure of *Lb. reuteri* GTF180-ΔN and sequence alignment of these two enzymes. The model was then checked for main chain conformations using the VADAR web server [46]. The geometry analysis showed that less than 4 % of the Φ-Ψ plots fell into the generously allowed and disallowed regions of the Ramachandran plot. Most of the residues which were in the generously allowed and disallowed regions are components of the loop regions in which there were major insertions and deletions relative to the template. On the basis of the 3D model built for parental DSR-S vardel Δ4N, we also generated a 3D model for the D460M:H463Y:T464M:S512C mutant, of which the linkage specificity was the most divergent from that of the parental enzyme. The overall protein folding of DSR-S vardel Δ4N and its mutant is shown Figure S1. Consistent with the GTF180-ΔN structure, the three domains, A, B and C were constituted by non-continuous sequence regions. In domain A, the (β/α)_8_-barrel catalytic domain is composed of three stretches of amino acids (364-614, 745-880 and 917-959). Domain B is inserted between helix α1 and strand β8 and also consists of three stretches (296-364, 880-917 and 959-973) and domain C is composed by residues 614-745 and forms the central part of the U-shaped protein.

### Description of the catalytic site, docking of substrate, and putative products

**Figure 5:**
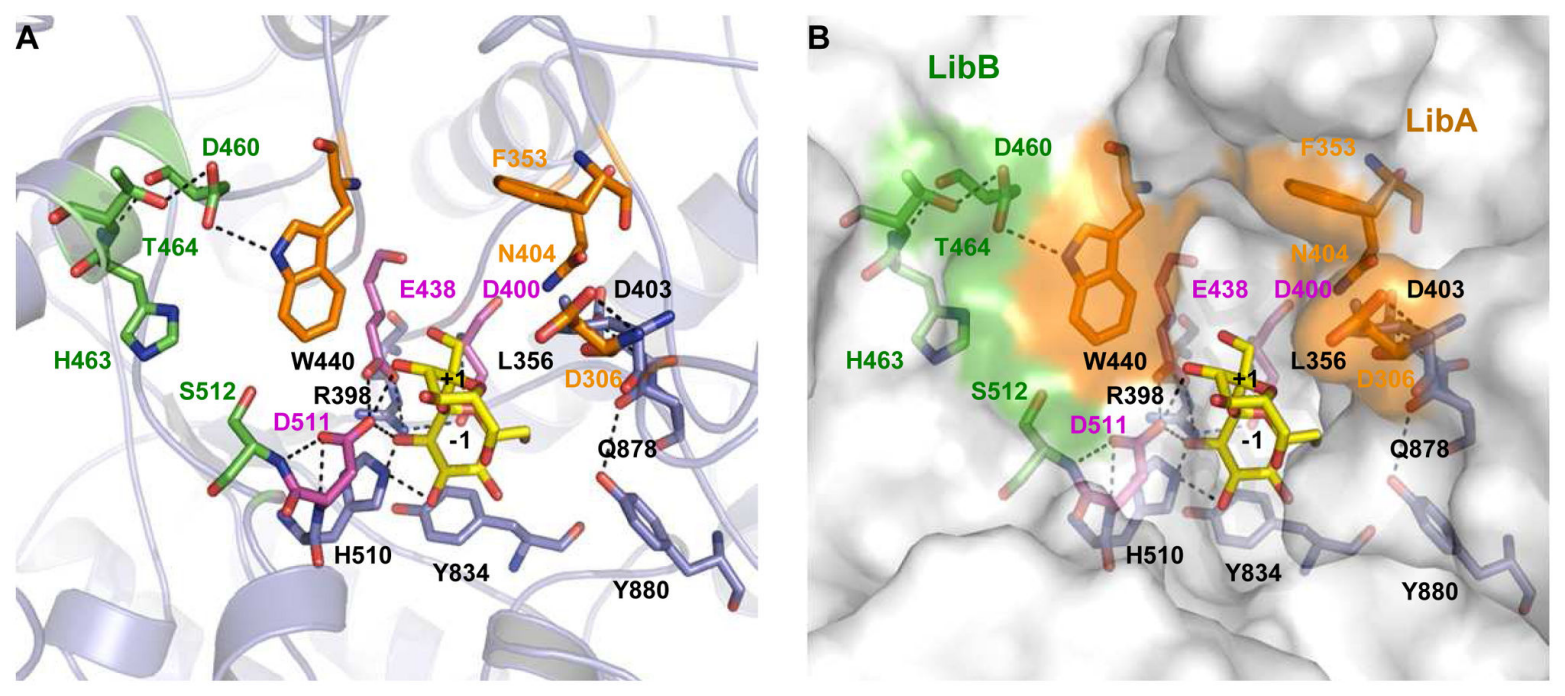
View of substrate binding in the active site of DSR-S vardel Δ4N. (A) Molecular docking of sucrose in the active site of DSR-S vardel Δ4N. (B) Overview of the positions of mutations affecting DSR-S vardel Δ4N linkage specificity. A docked sucrose molecule is shown for reference. Targeted residues are in orange (LibA) and green (LibB).

The 3D-model of DSR-S vardel Δ4N with sucrose bound in the catalytic site was used to describe binding interactions with the glucosyl ring at subsite -1 and the fructosyl ring at subsite +1 (Figure 5A). The topology of the enzyme active site appears to be a shallow pocket with the catalytic residues, namely the acid/base E438, the nucleophile D400 and the transition state stabilizer D511 (E1063, D1025 and D1136 in GTF180-ΔN, respectively), located at the bottom. The glucosyl moiety stacks onto Y834 in subsite -1 and establishes hydrogen bonding interactions with H510, D511 and Y880, while the β-D-fructosyl moiety interacts mostly with D511 in subsite +1. These residues are conserved in GTF180-ΔN.

The 3D-model of DSR-S vardel Δ4N was used to locate the eight amino acid positions targeted for randomization (Figure 5B). All belong to the first shell of amino acid residues less than 20 Å away from catalytic residues, residue S512 being the nearest as it is adjacent to catalytic D511. All the targeted positions are thus appropriately located to be involved in the binding of sucrose and oligosaccharides in the catalytic site. This is in agreement with the data obtained from the library analysis and kinetic characterization of the seven mutants with altered specificity.

**Figure 6:**
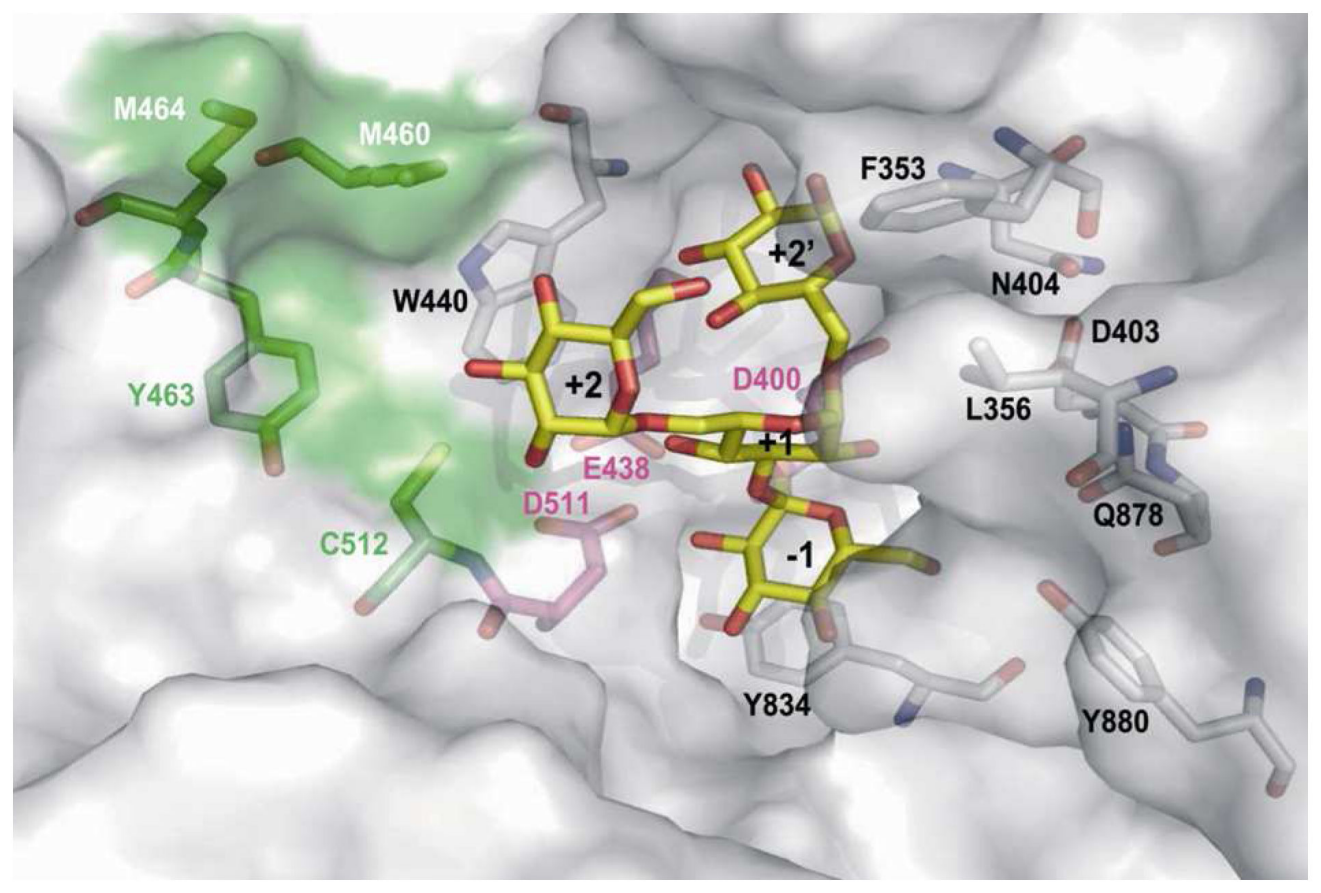
Molecular docking of α-D-Glcp-(1→6)[α-D-Glcp-(1→3)]α-D-Glcp-(1→6)-D-Glc*p* tetrasaccharide in the active site of the D460M:H463Y:T464M:S512C mutant.

To investigate the influence of the mutations on acceptor recognition, we performed docking studies of the tetrasaccharide α-D-Glcp-(1→6)[α-D-Glcp-(1→3)] α-D-Glcp-(1→6)-D-Glc*p* in the 3D model of D460M:H463Y:T464M:S512C. This tetrasaccharide is a putative product of glucosyl transfer onto isomaltotriose with the formation of an α(1→3) linkage. The favored binding mode of this tetrasaccharide is shown in Figure 6. The glucosyl ring at subsite -1 binds in a very similar way to the glucosyl unit of sucrose. The glucosyl unit at subsite +1 was also found in the productive conformation with respect to the acid/base E438 for the formation of an α(1→3) linkage. The +2 subsite accommodates the non-reducing end of the isomaltotriose through van der Waals interactions with W440T.

The +2’ subsite is formed by a small pocket between loops carrying the catalytic residues D400 and E438. The topology of this pocket is also determined by amino acids F353 and N404, targeted in LibA. It is therefore plausible that the mutations at these two positions, F353W and N404G, affect binding pocket topology and thus acceptor recognition. The positions at which mutations affect the linkage specificity, as in the D460M:H463Y:T464M:S512C mutant, are located in close spatial proximity to W440 (+2 subsite). In particular, the H463Y mutation could improve the stacking platform allowing interaction with longer oligosaccharide chains and thereby favor their binding. The presence of a polar side chain at position 512 (serine in the parental enzyme or cysteine in the mutant) also appears to be required for establishing hydrogen bonding interactions with hydroxyl groups of the acceptor glucosyl unit bound at +2 subsite. This is consistent with the putative role of S512 in oligosaccharide binding, as suggested previously [11]. 

The mutations introduced at subsite +2 thus seem to promote productive binding of α(1→6) linked glucooligosaccharides in the active site and favor the α(1→3) branching transglucosylation of glucan chains. However, the branching pattern of products synthesized by mutant D460M:H463Y:T464M:S512C will have to be characterized in more details to allow a better understanding of how the protein accommodates oligosaccharide molecules in the catalytic pocket, and how this differs from the parental DSR-S vardel Δ4N.

## Conclusions

Herein, we demonstrate the value of combinatorial engineering for investigating the relationships between structure and specificity of glucansucrases. Focusing on the catalytic domain of DSR-S vardel Δ4N dextransucrase, we identified key positions involved in the control of its specificity. In particular, this is the first time that the role of the peptide sequence ^460^DYVHT^464^ was investigated and demonstrated to be essential for α(1→3) linkage formation. It appears that modifications of acceptor binding at subsite +2 are responsible for the promotion of α(1→3) linkage formation. We also describe the design of catalytic pocket-directed libraries of mutants, combined with efficient screening protocols. This allowed the isolation of eighty-two variants producing HMW α-glucans with various proportions of α(1→3) linkages. These libraries of mutants are novel enzymatic tools for glycodiversification.

## Supporting Information

Table S1 **Primers and degenerated oligonucleotides used for combinatorial site-directed mutagenesis of dsr-s vardel Δ4N. Underlined nucleotides correspond to the introduced restriction sites.**
(DOCX)

Figure S1 **Overall structure of GS catalytic domain.** (**A**) **3D-model of DSR-S vardel Δ4N catalytic domain**. (**B**) **Catalytic domain of *Lactobacillus reuteri* N-terminally truncated glucansucrase GTF180 (PDB accession code: 3KLK)**.
(DOCX)
