# Supplementary material for: Combinatorial Engineering of Dextransucrase Specificity
Source: PLoS One. 2013 Oct 18;8(10):e77837. doi: 10.1371/journal.pone.0077837 (PMC3799614; doi:10.1371/journal.pone.0077837)
Supplement: Table S1 — Primers and degenerated oligonucleotides used for combinatorial site-directed mutagenesis of dsr-s vardel Δ4N. Underlined nucleotides correspond to the introduced restriction sites. (DOCX) [file pone.0077837.s001.docx]

**Supporting information**

**Table S1.** Primers and degenerated oligonucleotides used for combinatorial site-directed mutagenesis of dsr-s vardel Δ4N. Underlined nucleotides correspond to the introduced restriction sites.

| **Inverse PCR** |  |
| --- | --- |
| for_DSR-S_dAatII | 5’-TCCGTTGATAGTAATGACGTCTTAACAAGTGTTCGCTAT-3’ |
| rev_DSR-S_dAatII | 5’-ATAGCGAACACTTGTTAAGACGTCATTACTATCAACGGA-3’ |
| **Cassette amplification** |  |
| for_DSR-S_Alpha3 | 5’-CAAAGTACAGAGTGGTTAAAGGATGCAATG-3’ |
| rev_DSR-S_Alpha8 | 5’-ATACCTTCAGTACGCGTCTCAGATGTTC-3’ |
| **Libraries construction** |  |
| **LibA** |  |
| OligoD306X | 5’-GAAGATATGAGCAAT**NNS**CAT*C*T*G*CA*G*AATGGCGCATTA-3’ |
|  | *Pst*I |
| OligoF353X | 5’-AATTCAAAAGGTGGT**NNS**GAATTGTTG*C*TAGCCAATGAC-3’ |
|  | *Nhe*I |
| OligoN404X | 5’-GTAGATGCAGTCGAC**NNS**GTGGATGC*A*GAT*C*TGTTACAAATT-3’ |
|  | *Bgl*II |
| OligoW440X | 5’-CATCTTTCAATT*C*T*A*GAAGAT**NNS**AGTCACAATGATCCT-3’ |
|  | *Xba*I |
| **LibB** |  |
| OligoXXX | 5’-CAAGAAGCAATCA*G*TTAACCATGGAT**NNS**TATGTG**NNSNNS**CAATTAATCTGGTCTCTA-3’ |
|  | *Hpa*I |
| OligoS512X | 5’-AGCTTTGTACGTGC*G*CACGAC**NNS**GAAGTGCAAACG-3’ |
|  | *Fsp*I |
| **Nested PCR** |  |
| for_K7a3_nted | 5’-CCACAGTGGAATGAAACTAGTGAAGATATG-3’ |
| rev_K7a8_nted | 5’-ATGGCATCTTTACCATAGCGAACACTT-3’ |
