# Supplementary material for: Combinatorial Engineering of Dextransucrase Specificity
Source: PLoS One. 2013 Oct 18;8(10):e77837. doi: 10.1371/journal.pone.0077837 (PMC3799614; doi:10.1371/journal.pone.0077837)
Supplement: Figure S1 — Overall structure of GS catalytic domain. (A) 3D-model of DSR-S vardel Δ4N catalytic domain. (B) Catalytic domain of Lactobacillus reuteri N-terminally truncated glucansucrase GTF180 (PDB accession code: 3KLK). (DOCX) [file pone.0077837.s002.docx]

**Supporting information**


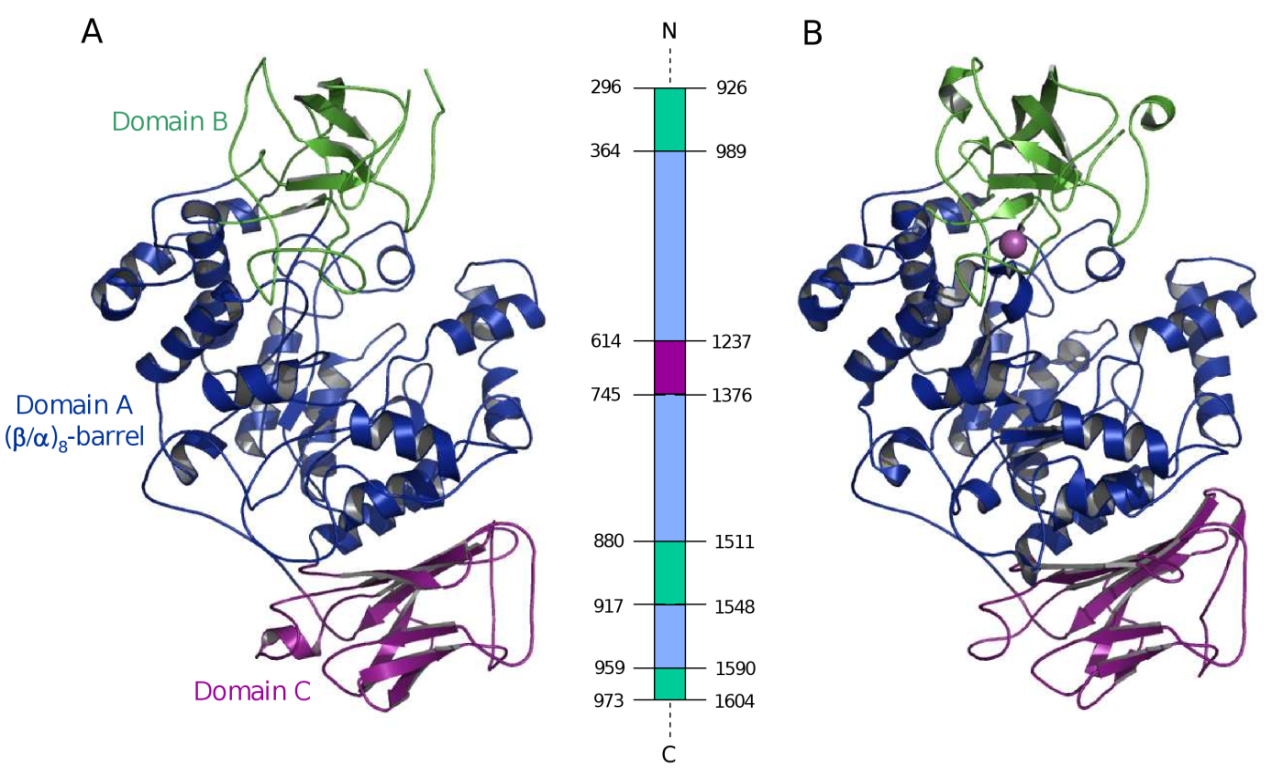


Figure S1. Overall structure of GS catalytic domain. (A) 3D-model of DSR-S vardel Δ4N catalytic domain. (B) Catalytic domain of *Lactobacillus reuteri* N-terminally truncated glucansucrase GTF180 (PDB accession code: 3KLK).
